# Dynamic causal modeling with neural fields

**DOI:** 10.1016/j.neuroimage.2011.08.020

**Published:** 2012-01-16

**Authors:** D.A. Pinotsis, R.J. Moran, K.J. Friston

**Affiliations:** The Wellcome Trust Centre for Neuroimaging, University College London, Queen Square, London WC1N 3BG, UK

**Keywords:** Neural field theory, Spectral analysis, Dynamic causal modeling, Connectivity, Neural mass models, Electrophysiology

## Abstract

The aim of this paper is twofold: first, to introduce a neural field model motivated by a well-known neural mass model; second, to show how one can estimate model parameters pertaining to spatial (anatomical) properties of neuronal sources based on EEG or LFP spectra using Bayesian inference. Specifically, we consider neural field models of cortical activity as generative models in the context of dynamic causal modeling (DCM). This paper considers the simplest case of a single cortical source modeled by the spatiotemporal dynamics of hidden neuronal states on a bounded cortical surface or manifold. We build this model using multiple layers, corresponding to cortical lamina in the real cortical manifold. These layers correspond to the populations considered in classical (Jansen and Rit) neural mass models. This allows us to formulate a neural field model that can be reduced to a neural mass model using appropriate constraints on its spatial parameters. In turn, this enables one to compare and contrast the predicted responses from equivalent neural field and mass models respectively. We pursue this using empirical LFP data from a single electrode to show that the parameters controlling the spatial dynamics of cortical activity can be recovered, using DCM, even in the absence of explicit spatial information in observed data.

## Highlights

► Neural field models are used as generative models in dynamic causal modelling (DCM). ► Relation between neural field and mass models is elucidated. ► Neural field models provide better explanations of empirical data. ► Bayesian model selection can distinguish between field and mass formulations. ► Spatial dynamics parameters obtained in the absence of explicit spatial information.

## Introduction

This paper is about estimating the spatiotemporal dynamics on cortical manifolds that produce electrophysiological measurements. Its focus is on recovering estimates of the underlying neuronal connectivity that shapes the spatial scale of neuronal fluctuations subtending observed data. In particular, we are interested in how changes in lateral or horizontal connections might be expressed in the time domain, in terms of spectral responses. There have been rapid advances in our ability to observe the spatiotemporal organization of cortical activity at a mesoscopic scale: modern two-dimensional multi-electrode arrays allow for the simultaneous recording of extracellular activity from a large number of neurons. These arrays yield important information about pairwise and higher order correlations, and have been used to study neural activity in visual (Kelly et al., 2007) and motor cortex (Hochberg et al., 2006). Similar samples of distributed neuronal activity have been obtained with silicon probes (Buzsaki and Kandel, 1998). Voltage-sensitive optical imaging also offers spatially resolved *in vivo* measurements of distributed neural activity over large areas of the cortex (Arieli et al., 1995; Zepeda et al., 2004). Finally, optical imaging has been used in conjunction with electrophysiological recordings to address the spatiotemporal organization of cortical activity; such as the nature of the coupling between the firing of individual neurons and population dynamics.

Both high density electrophysiological recordings and voltage-sensitive optical imaging furnish data with high spatial resolution; however, the spatial features of the data are not always fully exploited. For example, multi-electrode arrays are often used to isolate the action potentials of single neurons to focus on relevant spike rates. In this paper, we consider observation or generative models that include the spatial features of cortical dynamics that underlie observed signals. Furthermore, we demonstrate that the spatial aspects of cortical activity can be manifest, and therefore estimated from, temporal responses that are not spatially resolved; for example, spectra measured with a single electrode: this recovery of spatial parameters from purely temporal information has been established by Robinson and colleagues (Robinson et al., 2004; van Albada et al., 2007, 2010). This ill-posed inverse problem is here finessed with the use of informed generative models of the sort used in dynamic causal modeling. For simplicity, we restrict this paper to the analysis of a single cortical source or local manifold. In future papers, we will generalize the neural field model described here to provide a dynamic causal model of multiple, distributed cortical sources that are coupled via extrinsic connections.

Dynamic Causal Modeling (DCM) allows for the comparison and estimation of biophysically plausible models of fMRI, EEG, MEG and LFP data (David et al., 2006; Friston et al., 2003; Penny et al., 2010). DCM calls on an underlying generative model to predict important features of observed data. To date, DCMs for electrophysiological data have been based largely on neural mass models, which use point sources (e.g., equivalent current dipoles) and preclude spatially extended dynamics. This means the spatiotemporal aspects of cortical activity are not modeled explicitly. Here, we try to extend the DCM approach to endow the underlying model with spatial structure of the sort seen in real cortical architectures. This entails the use of neural fields as generative models of cortical activity.

### Neural mass and field models

Neural mass models are a particular case of neural fields, where the states of populations of neurons are functions of time only. Such models can generate temporal responses from one or several interconnected populations and have been used successfully to explain empirical electrophysiological data from local field potentials (LFP) and EEG/MEG (see e.g. Kiebel et al., 2009; Lopes da Silva et al., 1974; Lumer et al., 1997; Liley et al., 1999; Moran et al., 2007; Riera et al., 2007; Steriade and Deschenes, 1984; Valdes et al., 1999; van Rotterdam et al., 1982). To date, neural mass models have been based upon point sources and formulated using ordinary differential equations (ODEs). A key challenge in this area is to model observed signals as being generated by continuous and spatially distributed neuronal activity, of the sort observed directly using high density multi-electrode arrays and optical imaging. Here, we address this challenge using neural field models.

Neural fields model current fluxes as continuous processes on the cortical manifold, using partial differential equations (PDEs) (please see Deco et al., 2008 for a review and also Atay and Hutt, 2006; Breakspear et al., 2006; Bressloff, 1996,2001; Coombes et al., 2003, 2007; Coombes, 2005; Freeman, 2003,2005; Ghosh et al., 2008a,b; Golomb and Amitai, 1997; Jirsa and Haken, 1996; Jirsa and Kelso, 2000; Jirsa, 2009; Lopes da Silva and Storm van Leeuwen, 1978; Liley et al., 2002; Nunez, 1995,1996; Nunez and Srinivasan, 2006; O'Connor and Robinson, 2005; Qubbaj and Jirsa, 2009; Robinson et al., 2001, 2002, 2003, 2004, 2005; Robinson, 2006; Rennie et al., 2000; Roberts and Robinson, 2008; Rowe et al., 2004). The key advance that neural field models offer, over conventional neural mass models, is that they embody spatial parameters (like the density and extent of lateral connections). This means, in principle, one can infer the spatial parameters of cortical infrastructures generating electrophysiological signals (and infer changes in those parameters over different levels of an experimental factor) from empirical data. This rests on modeling responses not just in time but also over space. Clearly, to exploit this sort of model, one needs to measure the temporal dynamics of observed cortical responses over different spatial scales; for example, with high-density recordings, at the epidural or intracortical level. However, as we will see later, the impact of spatially extensive dynamics is not restricted to expression over space but can also have profound effects on temporal (e.g., spectral) responses at one point (or averaged locally over the cortical surface). This means that neural field models may also have a key role in the modeling of non-invasive electrophysiological data that does not resolve spatial activity directly.

### Neural field models and steady-state responses

Steady-state (or ongoing) activity spectra, associated with neural fields, have been studied in the context of models of the whole cortex; e.g.(Jirsa, 2009). Robinson and colleagues (Robinson, 2006) have developed a neurophysiologically grounded field model of corticothalamic activity, which has proven successful in reproducing several properties of empirical EEG signals; such as the spectral peaks seen in various sleep states and seizure activity. We pursue this approach but focus on a local source (patch or manifold, e.g., a cytoarchitectonic area), as opposed to the global dynamics of the corticothalamic system. Technically, the spectra summarizing the response of cortical sources to ergodic fluctuations can be defined in terms of transfer functions that depend on spatial and temporal parameters of the underlying cortical dynamics (Freeman, 1972; Nunez, 1995; Robinson, 2003; Robinson et al., 2001). In (Pinotsis and Friston, 2011), we derived the transfer function for a source described by a neural field equation (under an adiabatic approximation for fast postsynaptic filtering). Here, we dispense with the adiabatic approximation and derive the appropriate linear algebra for a cortical source that comprises multiple layers. In contrast to our earlier work, this allows us to consider synaptic processing with time scales that correspond to fluctuating inputs and propagation effects.

This paper comprises four sections. In the first, we present an overview of neural field models, starting from the basic principles of dynamical systems. We introduce the underlying concepts, in particular the role of spatially extended connectivity kernels and their associated transfer functions. This section then turns to predicted responses under steady-state assumptions; namely, the predicted cross-spectra observed among channels. The key idea described in this section is that one can summarize the mapping from fluctuating inputs (that can be experimental or spontaneous) to observed responses with a transfer function. Crucially, this transfer function depends upon the connectivity kernels encoding lateral neuronal interactions among different cortical layers and associated synaptic dynamics. In the second section, we turn to a particular example afforded by the Jansen and Rit (1995) model of a cortical source. We convert this neural mass model into a neural field model, using the framework established in the previous section. We illustrate some of the basic properties of this model before turning to its Bayesian formulation in the context of dynamic causal modeling, The third section reviews model inversion with a special focus on inverting models of complex data features, such as the complex cross-spectra predicted by the neural field models of the previous sections. In the final section, we apply the model to simulated and empirical LFP data acquired from the auditory cortex of rats. Our special interest here is in establishing face validity (of the model and its inversion) and comparing the predictions of neural field and homologous neural mass models; both in terms of the conditional estimates of the underlying spatial and synaptic parameters; and how changing these parameters affect the spectra predicted. We conclude with a discussion of further work using; (i) multiple cortical sources and (ii) multiple channels that sample a single source.

## Neural field models

In this section, we develop the basic formalism for neural field models that will be used in subsequent sections. We will start with a simple model that has no spatial attributes and develop this model into a neural field model. Our starting point is a dynamical formulation of any system with hidden states: a dynamical system is specified by two equations, namely, the state and observer equations given (in our case) by

(1) $$\begin{array}{c} \dot{V}(t)=f(V,U,\theta )\\ Y(t)=L(V,\theta ) \end{array}$$

where V(t)∈R and U(t)∈R are vectors of hidden state variables and inputs. Here, we use *θ* to denote the parameters of the model. Dynamical systems of the kind described by Eq. (1) are used extensively in engineering and applied sciences and are the basis of so-called state space models (Valdes-Sosa et al., 2009). Such models are used in the analysis of neuroimaging data, particularly in the context of Dynamic Causal Modeling (DCM). Many generative models can be cast in the form of Eq. (1) and include neural mass models, such as the Jansen and Rit model (1995).

Neural mass models prescribe an appropriate vector-valued function *f* describing the dynamics (flow) of *hidden* neuronal states, V(t)∈R. For example, in event-related potential (ERP) studies, these states correspond to the average depolarization of neuronal populations; e.g., pyramidal neurons. The term ‘hidden’ follows from the fact that neuronal states are not measured directly but are inferred from the observed responses in sensor space: the hidden states are mapped to sensor space through a spatial forward model (usually involving a lead field) modeled by *Y* = *L*(*V*, *θ*) where *Y*(*t*) denotes observed data. In EEG and MEG this mapping is usually linear *Y* = *L* ⋅ *V* and specified with a gain matrix of lead-fields, *L*(*φ*) with unknown spatial parameters, *φ* ⊂ *θ*, such as source location and orientation. Generally, this matrix rests upon the solution of a well-posed electromagnetic forward model. For invasive LFP recordings (that are obtained directly from neuronal sources) the gain matrix may simply encode electrode-specific gains.

In Eq. (1), the vector-valued function V(t)∈R depends on time only. In other words, the hidden states appearing in dynamic causal models are usually only functions of time and do not depend on the position on the cortical manifold. Although, neural mass models can describe patterns in sensor space, the spatial attributes of these patterns result from the coupling among states at different points in source space and not from hidden states that are functions of the source space itself. This means that neural mass models are not constrained by the local topography of lateral synaptic connections, in particular the local patchy distribution of connections in the brain that are a hallmark of functional specialization (Zeki, 1990). Functional specialization demands that cells with common functional properties are grouped together. This architectural constraint necessitates both convergence and divergence of cortical connections, of the sort that can be modeled with a neural field model. To model these spatial aspects one needs partial differential or integrodifferential equations that accommodate lateral interactions and model dynamics that play out on a spatially extended cortical manifold: see (Deco et al., 2008), for a review of mean-field, neural mass and field models.

In what follows, we assume that a cortical source can be approximated by *n* layers on a homogeneous Euclidean manifold, each point of which can be determined by some coordinates *x* and *t*. In the context of neural field theory, the *n* × 1 vector V(t)∈R pertaining to the hidden neuronal state of each layer is replaced by V(x,t)∈R; namely, a vector depending on both time and space. The dynamics of cortical sources now conform to integrodifferential equations, such as the Wilson–Cowan or Amari equations. Systems of such equations can serve as generative models since they can be cast in the form of Eq. (1). In particular, assuming synaptic filtering of the first-order (see next section), a system of neural field equations describing *n* interacting layers can be written in the following general form

(2) $$\begin{array}{c} \dot{V}=-BV+D⊗F\circ V+G\circ U\\ Y=L\cdot V\\ V=\left[\begin{array}{c} v_{1}(x,t)\\ \vdots \\ v_{n}(x,t) \end{array}\right] \end{array}$$

where *V*(*x*, *t*) is a vector of depolarizations and ⊗ denotes a spatiotemporal convolution operator

(3) $$D⊗Q=\iint D\left(x-x^{\prime },t-t^{\prime }\right)\cdot Q(x^{\prime },t^{\prime })dx^{\prime }dt^{\prime }\text{.}$$

In the above equations, *D*(*x*, *t*) is a *n* × *n* smooth (analytic) matrix-valued connectivity function or kernel, $F:R^{n}\to R^{n}$ is a nonlinear mapping from postsynaptic depolarization to presynaptic firing rates at each point on the cortical manifold and *B* is a *n* × *n* matrix encoding average synaptic decay rates. In short, Eq. (2) says that the rate of change of voltage in each layer comprises three terms; the first is a simple decay, the second is due to presynaptic inputs from other parts of the cortical manifold and the final part is due to external inputs, where $G:R^{n}\to R^{n}$ maps the inputs to the motion of hidden states. It is the second component, involving the convolution with the connectivity kernel *D*(*x*, *t*) that embodies lateral interactions over the cortical manifold. In terms of the observer function, the linear mapping from hidden states to observed signal now becomes a *m* × *n* matrix function of source space *L*(*x*, *φ*) encoding the contribution of the *n* hidden states to each of *m* sensors; such that the response that the *i*-th electrode is *y*_*i*_(*t*) = ∫ *L*_*i*_(*x*, *φ*) ⋅ *V*(*x*, *t*)*dx*. This means that we have to specify the gain matrix as a function of source space. This will become relevant later when we derive expressions for predicted responses in sensor or channel space.

In the next section, we will consider particular examples of the equations above. In general, the integrodifferential nature of Eq. (2) precludes a full analytical treatment. Therefore, one usually resorts to suitable approximations to obtain solutions. Solutions of such equations include spatially and temporally periodic patterns beyond Turing instabilities; for example, localized regions of activity such as bumps and travelling waves, see Pinto and Ermentrout (2001), Coombes (2005), Laing and Troy (2003), Hutt et al. (2003), Atay and Hutt (2006), Laing and Chow (2001) and Rubin and Troy (2004). One well-known approach to solving Eq. (2) is to take its Fourier transform. In this context, the spatiotemporal convolution in Eq. (3) yields partial differential equations (PDEs) in the form of wave equations; assuming that the Fourier transforms of the connectivity kernels are rational and well-behaved (Jirsa and Haken, 1997). For notational simplicity, we will retain the same name for variables in the time and frequency domain and represent the Fourier transform of a function implicitly through its arguments such that *D*(*k*, *ω*) is the Fourier transform of *D*(*x*, *t*).

We will return to Fourier transforms in the examples below; however, the approach we pursue starts with assuming that the system is at steady-state^1^ and is perturbed by some exogenous input or fluctuations. If the input is stationary (ergodic), then the ensuing activity corresponds to steady-state activity. If the input is manipulated experimentally (e.g., a stimulus), then the solutions to Eq. (2) model induced or evoked responses about the steady-state solution. In either case, we can obtain an expression for the transfer function of the system of neural fields defined by Eq. (2) by linearizing around a steady-state. This allows us to express the system's spectral responses in terms of its key architectural parameters pertaining to postsynaptic filtering and the local connectivity patterns entailed by *D*(*x*, *t*). This means one can, in principle, estimate the temporal and spatial parameters of cortical sources, given observed spectral responses. We now consider this in a bit more detail.

### Spectral formulations

The input *U*(*t*) appearing in Eq. (1) can encompass designed experimental effects; namely, functions that encode experimental manipulations or context. In DCM for event-related potentials these inputs are treated as known (or parameterized) functions of time and estimates of parameters encoding the connectivity among sources are based upon measuring the response of the system to these inputs. This approach should be contrasted with other methods, such as DCM for steady-state responses, structural equation modeling and multivariate autoregressive modeling, which consider the inputs as stationary random fluctuations with known (or parameterized) covariance. In DCM, connectivity is usually parameterized in terms of coupling parameters *D* ⊂ *θ* and other physiological parameters, such as those controlling firing rates and postsynaptic filtering. The coupling parameters or *effective connectivity* are either intrinsic to each (point) source or extrinsic (coupling different sources). In the case of neural field models, the function *U*(*t*) is replaced by *U*(*x*, *t*). This means the input is an explicit function of both space and time. Furthermore, coupling is now parameterized by coupling kernels *D*(*x*, *t*) that are functions of source space (both within and between sources). This means one can infer the spatial parameters of cortical infrastructures generating electrophysiological signals (and infer changes in those parameters over different levels of an experimental factor) from electrophysiological measurements.

We now focus on a mathematical description of the effect of external input or fluctuations driving a cortical source described by Eq. (2). As in Pinotsis and Friston (2011) (see also Grindrod and Pinotsis, 2011), we assume that the system is perturbed around a spatially homogeneous steady-state *V*_0_, that satisfies *f*(*V*_0_) = 0. In other words, we substitute

(4) $$V(x,t)=V_{0}+P(x,t)$$

into Eq. (2) and expand *F* ∘ *V* around *V*_0_ to obtain a first-order expression for the perturbations *P*(*x*, *t*) around the fixed point

(5) $$\begin{array}{c} \dot{P}=-BP+D⊗\gamma P+U\\ \gamma =\partial F(V_{0})/\partial V\text{.} \end{array}$$

Here, *γ* is the Jacobian of the nonlinear mapping evaluated at *V*_0_ and can be thought of as the gain of some depolarization-firing function (see below). For simplicity, we have assumed

(6) $$G\circ U=U=\left[\begin{array}{c} u_{1}\\ \vdots \\ u_{n} \end{array}\right]\text{.}$$

Here, *u*_*i*_ = *u*_*i*_(*x*, *t*) : *i* ∈ 1, …, *n* are the external inputs to each source. Taking the Fourier transform of Eq. (5), we obtain

(7) $$\begin{array}{c} \dot{P}+BP-D⊗\gamma P=U\\ \Rightarrow \\ \left(i\omega I_{n}+B-D(k,\omega )\gamma )\cdot P(k,\omega )=U(k,\omega \right) \end{array}$$

where *I*_*n*_ is the *n* × *n* identity matrix and *U*(*k*, *ω*) is a *n* × 1 vector containing the two dimensional Fourier transforms of the input

(8) $$U_{i}(k,\omega )=\iint U_{i}(x,t)e^{-ikx-i\omega t}dxdt\text{.}$$

The Fourier transforms *P*(*k*, *ω*) and *D*(*k*, *ω*) are *n* × 1 and *n* × *n* matrices containing the transforms of the perturbations *P*(*x*, *t*) and (delay) kernels *D*(*x*, *t*) parameterizing the connectivity between layers *i* and *j*, respectively. Eq. (7) implies that the matrix transfer function of the system, namely the mapping from inputs to perturbations of the hidden states is given by

(9) $$T(k,\omega )={\left(i\omega I_{n}+B-D(k,\omega )\gamma \right)}^{-1}\text{.}$$

This transfer function allows us to express the perturbations in terms of the inputs, namely

(10) $$\begin{array}{c} P(x,t)=\iint T(x-x^{\prime },t-t^{\prime })U(x^{\prime },t^{\prime })dx^{\prime }dt^{\prime }\\ \Leftrightarrow \\ P(k,\omega )=T(k,\omega )U(k,\omega )\text{.} \end{array}$$

The ensuing cross-spectra can be derived easily from the transfer function matrix *T*(*k*, *ω*). Crucially, under stationarity assumptions, the temporal form of the inputs is not required to predict the cross-spectra of the perturbations to hidden states: all we require is their cross-spectrum *g*_*U*_(*k*, *ω*) = *U*(*k*, *ω*)U*(*k*, *ω*), where * denotes the complex conjugate matrix transpose. Also, assuming that the inputs to different sources are independent, namely that *u*_*i*_(*k*, *ω*)*u*_j_*(*k*, *ω*) = 0 : *i* ≠ *j* the cross-spectrum of the inputs is a diagonal matrix with off diagonal (cross-spectral density) entries of zero. Finally, the complex cross-spectra, at a spatial frequency *k* and temporal frequency *ω*, of perturbations in source and sensor space are given by

(11) $$\begin{array}{c} g_{P}(k,\omega )=T(k,\omega )g_{U}(k,\omega )T{\left(k,\omega \right)}^{*}\\ g_{Y}(\omega ,\theta )=\int L(k)g_{P}(k,\omega )L^{*}(k)dk\text{.} \end{array}$$

Here, *L*(*k*) ≜ *L*(*k*, *φ*) is the (spatial) Fourier transform of the lead fields that weights the contribution of each (*k*-th) spatial frequency to the observed spectra. Eq. (11) reflects the fact that the lead fields act as spatial filters, selectively sampling temporal frequencies that are expressed in the range of spatial frequencies the lead field can see. An ideal sensor would be sensitive to all frequencies therefore the integral in Eq. (11) would weight contributions from all spatial frequencies equally, with *L*(*k*) = 1*.* However, a real sensor will only be sensitive to a certain range of frequencies. For example, if the lead field has a narrow spatial support (e.g., LFP electrodes), its Fourier transform will be broad and it will be sensitive to a wide range of spatial frequencies. Conversely, when the lead field is broad (e.g., non-invasive MEG sensors), only low spatial frequencies will contribute to the observed cross-spectra.

### Summary

In short, Eq. (11) couples the observed spectral responses of the system to its *spatial* as well as its temporal properties (see e.g. Jirsa, 2009; Nunez, 1995; Rennie et al., 2000; Robinson, 2006). These properties are encoded in the transfer function *T*(*k*, *ω*), through the underlying connectivity functions *D*(*k*, *ω*). We now unpack some of this general formalism and see how it can be used to model a cortical source comprising three layers on a bounded manifold. It should be noted that the three layer source used in this paper is one of many models to which our approach can be applied: we focus on the Jansen and Rit model for illustration purposes and because it is a widely used model that has been biophysically validated.

## The Jansen and Rit model

In this section, we provide a brief review of the well-known Jansen and Rit neural mass model (Jansen and Rit, 1995) and transcribe it into a neural field model using the equations of the previous section. In the Jansen and Rit model, each cortical source is modeled with three subpopulations: excitatory spiny stellate input cells, inhibitory interneurons and deep excitatory output pyramidal cells (for classical approaches to modeling such populations with neural fields, see e.g. Amari, 1977; Freeman, 1972; Nunez, 1995; Wilson and Cowan, 1973). For simplicity, in this paper we consider a single source, noting that extensions to multiple sources involve adding extrinsic (between-source) connections or kernels (see discussion). The Jansen and Rit model is a particular instance of Eq. (1). However, the postsynaptic convolution of presynaptic inputs in the Jansen and Rit model is second-order. In other words, it is described by a second-order ODE or two first-order ODEs pertaining to voltage and current. This means that the left hand side of Eq. (1) is augmented with the second derivative of hidden (depolarization) states to give

(12) $$\begin{array}{c} \ddot{V}+2B\dot{V}=-B^{2}V+ABF\circ V+GU\\ Y=L\cdot V \end{array}$$

where *A* and *B* are the 3 × 3 matrices of synaptic parameters controlling the maximum postsynaptic responses and the rate-constants of postsynaptic filtering (cf, decay):

(13) $$\begin{array}{c} A=diag\left(m_{e},m_{i},m_{e}\right)\\ B=diag\left(\kappa_{e},\kappa_{i},\kappa_{e}\right)\\ G=\left[\begin{array}{c} \kappa_{e}m_{e}\\ 0\\ 0 \end{array}\right]\text{.} \end{array}$$

**Fig. 1:**
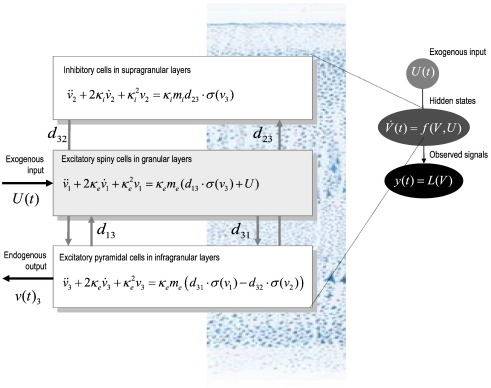
*Equations of motion for a single source*. This schematic summarizes the equations of motion or state equations that specify a Jansen and Rit (1995) neural mass model of a single source. This model contains three populations, each loosely associated with a specific cortical layer. The second-order differential equations describe changes in hidden states (e.g., voltage) that subtend observed local field potentials or EEG signals. These differential equations effectively mediate a linear convolution of presynaptic activity to produce postsynaptic depolarization. Average firing rates within each sub-population are then transformed through a nonlinear (sigmoid) voltage-firing rate function *σ*(⋅) to provide inputs to other populations. These inputs are weighted by connection strengths.

As above F:R→R is a nonlinear mapping from depolarization to firing and U(t)∈R is the external input to each population. Note that there is only one input that enters the first (spiny stellate) population. Based on a canonical microcircuitry of intrinsic connections, the Jansen and Rit model prescribes the mapping F:R→R in terms of nonlinear firing rate functions of the depolarization in the three populations. Writing out Eq. (12) in full we have

(14) $$\begin{array}{c} {\ddot{v}}_{1}+2\kappa_{e}{\dot{v}}_{1}+\kappa_{e}^{2}v_{1}=\kappa_{e}m_{e}(d_{13}\cdot \sigma (v_{3})+U)\\ {\ddot{v}}_{2}+2\kappa_{i}{\dot{v}}_{2}+\kappa_{i}^{2}v_{2}=\kappa_{i}m_{i}d_{23}\cdot \sigma (v_{3})\\ {\ddot{v}}_{3}+2\kappa_{e}{\dot{v}}_{3}+\kappa_{e}^{2}v_{3}=\kappa_{e}m_{e}\left(d_{31}\cdot \sigma (v_{1})-d_{32}\cdot \sigma (v_{2})\right) \end{array}$$

where *v*_*i*_(*t*) : *i* = 1, 2, 3 denotes the expected depolarization in the *i*-th population (excitatory stellate, inhibitory population and excitatory pyramidal respectively) and *d*_*ij*_ · *σ*(*v*_*j*_) is the presynaptic input to the *i*-th population from the *j*-th. This is a sigmoid function *σ*(*v*_*j*_) of postsynaptic depolarization in the *j*-th population, multiplied by intrinsic connection strengths *d*_*ij*_ between the two populations (Jansen and Rit, 1995). See Fig. 1 for a schematic of this model.

### A Jansen and Rit neural field model

In the following, we extend the Jansen and Rit model for spatially extended sources on the cortical manifold. Our approach follows standard treatments of neural field models (see e.g. Coombes et al., 2007; Pelinovsky and Yakhno, 1996; Pinto et al., 1996; Robinson et al., 2001, 2003, 2005; Wilson and Cowan, 1973) and focuses on a model with realistic connection patterns among three neuronal populations. In doing this we hope to show how one can transcribe a neural mass into a neural field model; where the former can be regarded as the limiting case of the latter. To do this, we treat each of the three populations above as a separate layer on the cortical manifold. This means that depolarization $V(x,t)\in R^{3}$ now becomes a vector-field as opposed to a vector and the intrinsic connection strengths become connectivity kernels, *d*_*ij*_(*x*, *t*). The ensuing model can be written in the general form

(15) $$\begin{array}{c} \ddot{V}+2B\dot{V}=-B^{2}V+ABD⊗F\circ V+GU\\ Y=L\cdot V\text{.} \end{array}$$

Here, the spatiotemporal convolution term *D* ⊗ *F* includes the (delayed) presynaptic input arriving from all layers in the cortical manifold. Augmenting Eq. (14) with appropriate spatiotemporal convolutions, we obtain

(16) $$\begin{array}{c} \left({\ddot{v}}_{1}+2\kappa_{e}{\dot{v}}_{1}+\kappa_{e}^{2}v_{1}\right)(x,t)=\kappa_{e}m_{e}\left(\iint d_{13}(x-x^{\prime },t-t^{\prime })\sigma (v_{3}(x^{\prime },t^{\prime }))dx^{\prime }dt^{\prime }+U\right)\\ \left({\ddot{v}}_{2}+2\kappa_{i}{\dot{v}}_{2}+\kappa_{i}^{2}v_{2}\right)(x,t)=\kappa_{i}m_{i}\iint d_{23}(x-x^{\prime },t-t^{\prime })\sigma (v_{3}(x^{\prime },t^{\prime }))dx\prime dt^{\prime }\\ \left({\ddot{v}}_{3}+2\kappa_{e}{\dot{v}}_{3}+\kappa_{e}^{2}v_{3}\right)(x,t)=\kappa_{e}m_{e}\iint \left(d_{31}(x-x^{\prime },t-t^{\prime })\sigma (v_{1}(x^{\prime },t^{\prime }))-d_{32}(x-x^{\prime },t-t^{\prime })\sigma (v_{2}(x^{\prime },t^{\prime }))\right)dx^{\prime }dt^{\prime } \end{array}$$

where we assume the sigmoid firing rate function is:

(17) $$\sigma (v_{i})=\frac{1}{1+\exp(r(\eta -v_{i}))}\text{.}$$

Here, *r* and *η* are parameters that determine the shape of this sigmoid. In particular, *r* is synaptic gain and *η* is the postsynaptic potential at which the half of the maximum firing rate is achieved. Following the approach for the linearization of neural fields described above, we assume a solution to Eq. (15) of the form

(18) $$V(x,t)=\left[\begin{array}{c} v_{1}(x,t)\\ v_{2}(x,t)\\ v_{3}(x,t) \end{array}\right]=V_{0}+P(x,t)\text{.}$$

Taking the Fourier transform of Eq. (15) we obtain the frequency formulation of the Jansen and Rit neural field model

(19) $$\begin{array}{c} \ddot{V}+2B\dot{V}=-B^{2}V+ABD⊗F\circ V+GU\\ \Rightarrow \\ \left(-\omega^{2}I_{3}+2i\omega B+B^{2}-J(k,\omega ))P(k,\omega )=GU(k,\omega \right) \end{array}$$

where *J*(*k*, *ω*) is a 3 × 3 matrix incorporating the synaptic parameters, connectivity parameters (kernels) and gain matrix:

(20) $$\begin{array}{c} J(k,\omega )=ABD(k,\omega )\gamma \\ D=\left[\begin{array}{ccc} 0 & 0 & D_{13}(k,\omega )\\ 0 & 0 & D_{23}(k,\omega )\\ D_{31}(k,\omega ) & -D_{32}(k,\omega ) & 0 \end{array}\right]\\ \gamma =\partial \sigma (V_{0})/\partial V\text{.} \end{array}$$

Here *D*_*ij*_(*k*, *ω*) are the Fourier transforms of the connectivity kernels *d*_*ij*_(*x*, *t*). The matrix transfer function for the Jansen and Rit neural field model is then given by

(21) $$\begin{array}{c} T(k,\omega )={\left(2i\omega B+B^{2}-\omega^{2}I_{3}-J(k,\omega )\right)}^{-1}G\\ \Leftrightarrow \\ P(k,\omega )=T(k,\omega )U(k,\omega )\text{.} \end{array}$$

Combining Eqs. (21) and (11) we obtain the cross-spectra generated at the sensors by the Jansen and Rit neural fields described above.

(22) $$\begin{array}{c} g_{Y}(\omega ,\theta )=\int L(k)T(k,\omega )g_{U}(k,\omega )T{\left(k,\omega \right)}^{*}L{\left(k\right)}^{*}dk\\ T(k,\omega )={\left(2i\omega B+B^{2}-\omega^{2}I_{3}-J(k,\omega )\right)}^{-1}G\text{.} \end{array}$$

Note that for a single sensor, the cross-spectrum reduces to the (real) spectral density. Next, we turn to the particular forms of neural field models that are prescribed by the connectivity kernels.

### Connectivity kernels

In essence, Eq. (15) defines a class of neural field models that conform to the same intrinsic connectivity rules incorporated in the neural mass model; see Fig. 1 and Eq. (20). To fully specify a particular model in this class, we need to specify the functional form for the kernels, *d*_*ij*_(*x*, *t*). We will assume these kernels factorize into *d*_*ij*_(*x*, *t*) = *κ*_*ij*_(|*x*|)*δ*(*t* − *υ*_*ij*_|*x*|), where *υ*_*ij*_ is a transit time or the inverse of the speed *s*_*ij*_ with which neuronal firing propagates along connections. This form provides an explicit parameterization of conduction delays that will be exploited later, when using the field model as an observation model. We now need to specify the kernels *κ*_*ij*_(|*x*|), which describe the connectivity strength between layers *i* and *j* comprising the cortical source.

For each application, these kernels can be chosen to appropriately describe the spatial features of the underlying cortical infrastructure generating observed signals. Our aim here is to provide a link between the existing neural field literature and DCM techniques for Bayesian inference on cortical parameters based on observed spectra. We therefore adopt a common choice in the literature, which accounts for local excitatory and inhibitory interactions (Coombes et al., 2003; Jirsa and Haken, 1996; Wilson and Cowan, 1973). In particular, we assume that the functions *κ*_*ij*_ ∈ *K* have an exponential form, where, for a single source:

(23) $$2K=\left[\begin{array}{ccc} 0 & 0 & \alpha_{13}e^{-c_{13}|x|}\\ 0 & 0 & \alpha_{23}e^{-c_{23}|x|}\\ \alpha_{31}e^{-c_{31}|x|} & \alpha_{32}e^{-c_{32}|x|} & 0 \end{array}\right]\text{.}$$

The parameters *α*_*ij*_ and *c*_*ij*_ encode the strength (analogous to the total number of synaptic connections) and extent (spatial precision) of intrinsic connections between the cortical layers. In addition to its simplicity, the above form for the connectivity kernel also has the advantage that, by taking the Fourier transforms of both sides; Eq. (16) can be expressed in a *differential* form (see also Jirsa and Haken, 1996)

(24) $$\begin{array}{c} {\ddot{v}}_{1}+2\kappa_{e}{\dot{v}}_{1}+\kappa_{e}^{2}v_{1}=\kappa_{e}m_{e}(\mu_{1}+U)\\ {\ddot{v}}_{2}+2\kappa_{i}{\dot{v}}_{2}+\kappa_{i}^{2}v_{2}=\kappa_{i}m_{i}\mu_{2}\\ {\ddot{v}}_{3}+2\kappa_{e}{\dot{v}}_{3}+\kappa_{e}^{2}v_{3}=\kappa_{e}m_{e}\mu_{3}\\ {\ddot{\mu }}_{1}+2sc_{13}{\dot{\mu }}_{1}-s^{2}(\mu_{1xx}-c_{13}^{2}\mu_{1})=\alpha_{13}(s^{2}c_{13}\sigma (v_{3})+s\dot{\sigma }(v_{3}))\\ {\ddot{\mu }}_{2}+2sc_{23}{\dot{\mu }}_{2}-s^{2}(\mu_{2xx}-c_{23}^{2}\mu_{2})=\alpha_{23}(s^{2}c_{23}\sigma (v_{3})+s\dot{\sigma }(v_{3}))\\ {\ddot{\mu }}_{3}+2sc_{31}{\dot{\mu }}_{3}-s^{2}(\mu_{3xx}-c_{31}^{2}\mu_{3})=\alpha_{31}\left(s^{2}c_{31}(\sigma (v_{1})-\sigma (v_{2}))+s\left(\dot{\sigma }(v_{1})-\dot{\sigma }(v_{2})\right)\right)\text{.} \end{array}$$

In this form, the equations for presynaptic input *μ*_*ij*_ have the form of wave equations. For simplicity, we have assumed that the conduction velocity is the same for all connections; namely, *s*_*ij*_ = *s*. We also assumed that *α*_31_ = *α*_32_ and *c*_31_ = *c*_32_. Using PDE integration schemes (solvers), Eq. (24) can provide explicit predictions of depolarization in response to input, U(x,t)∈R. However, we will not pursue this here. Instead, we will use the spectra given by Eq. (11) to provide a generative model of observed cross-spectra generated by the underlying cortical sources. In this context, the matrix *J*(*k*, *ω*) is given by Eq. (20), where the connectivity transfer functions are (see Appendix)

(25) $$\begin{array}{l} D_{ij}(k,\omega )=\frac{\alpha_{ij}(c_{ij}+i\upsilon_{ij}\omega )}{c_{ij}^{2}-\upsilon_{ij}^{2}\omega^{2}+2i\upsilon_{ij}c_{ij}\omega +k^{2}}\\ \; \end{array}$$

and the gain matrix is

(26) $$\gamma_{ij}=\frac{\partial \sigma (v_{i}=0)}{\partial v_{j}}=\{\begin{array}{cc} \frac{re^{r\eta }}{{\left(1+e^{r\eta }\right)}^{2}} & i=j\\ 0 & i\neq j \end{array}\text{.}$$

Substituting these expressions into Eq. (20) allows us to express the predicted cross-spectra over channels, in terms of the parameters of our generative neural field model through Eq. (22). These parameters include the synaptic parameters associated with the Jansen and Rit neural mass model *θ* ⊂ {*m*_*i*_, *m*_*e*_, *κ*_*i*_, *κ*_*e*_, *r*, *η*} but now also include the spatial parameters $\theta \subset \{\alpha_{ij},c_{ij},\upsilon_{ij},\ell \}$ that encode intrinsic connections among the layers. In practice, to suppress redundancy in the parameterization of these neural field models, we express the spatial extent of horizontal connections and inverse velocity in terms of the radius of the cortical patch. This means that, without loss of generality, ℓ=1 and the inverse velocity becomes the (transit) time it takes for spikes to propagate from the centre of the patch to its boundary. We will pursue the inversion of these models in the next section. We conclude this section by looking at the typical spectra produced by a cortical source.

### Spectra of the Jansen and Rit neural field model

For purposes of illustration, we assume a single Jansen and Rit neural field and a realistic observation filter with a Gaussian form, which we parameterize in terms of its amplitude and width:

(27) $$L(x,\varphi )=\varphi_{1}\exp\left(-\frac{x^{2}}{\varphi_{2}}\right)\text{.}$$

In all the simulations and analyses below we used a small value of $\varphi_{2}=0.01\times \ell$, appropriate for a local field potentials electrode that are sensitive to a large range of frequencies (recall that ℓ=1 is the distance from the centre to the boundary of the cortical manifold). We also assumed that only pyramidal cells contribute to the observed signal. Under these assumptions, Eq. (22) implies

(28) $$\begin{array}{c} g_{Y}(\omega ,\theta )=\int L(k)T(k,\omega )g_{U}(k,\omega )T{\left(k,\omega \right)}^{*}L{\left(k\right)}^{*}dk\\ L(k)=\varphi_{1}\exp(-\varphi_{2}\pi^{2}k^{2})\\ T(k,\omega )=D_{31}(k,\omega )\gamma \kappa_{e}{}^{2}m_{e}{}^{2}{\left(\kappa_{i}+i\omega \right)}^{2}R^{-1}(k,\omega )\\ R(k,\omega )={\left(\kappa_{i}+i\omega \right)}^{2}\left(\kappa_{e}{}^{4}+4i\kappa_{e}{}^{3}\omega -4i\kappa_{e}\omega^{3}+\omega^{4}-\kappa_{e}{}^{2}\left(D_{13}(k,\omega )D_{31}(k,\omega )\gamma^{2}m_{e}{}^{2}+6\omega^{2}\right)\right)\\ -D_{23}(k,\omega )D_{32}(k,\omega )\gamma^{2}\kappa_{e}\kappa_{i}m_{e}m_{i}{\left(\kappa_{e}+i\omega \right)}^{2}\text{.} \end{array}$$

Under periodic boundary conditions, cortical activity can be viewed as a superposition of standing waves of various spatial frequencies, which are integer multiples of the frequency π/ℓ. Under these boundary conditions, Eq. (28) yields the following expression for the predicted (cross) spectra (see Robinson, 2003)

(29) $$g_{Y}(\omega ,\theta )\approx \frac{\pi }{\ell }\sum_{j}L\left(\frac{j\pi }{\ell }\right)T\left(\frac{j\pi }{\ell },\omega \right)g_{U}\left(\frac{j\pi }{\ell },\omega \right)T{\left(\frac{j\pi }{\ell },\omega \right)}^{*}L{\left(\frac{j\pi }{\ell }\right)}^{*}\text{.}$$

**Fig. 2:**
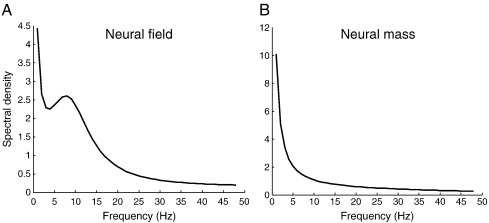
*Power spectra for a single source*. This figure shows the power spectra associated with a single source, whose dynamics conform to Eq. (24). The generative model is based upon Eq. (29) using the parameters described in the main text and Table 1. Panel A shows the spectral response using the mean field model, while panel B shows the equivalent predictions under a neural mass model: i.e. where the spectral prediction was obtained using exactly the same equations as for the neural field predictions but where the inverse conduction velocity or transit time was set to zero.

Fig. 2A shows the form of this spectral density using the expression for connectivity in Eq. (25), and the parameters in Table 1 for *j* = 1, …, 32 spatial frequencies. The crucial thing about this result is the alpha peak that results from a finite conduction velocity (Nunez, 1995; Robinson et al., 2001). This form is quite distinct from the equivalent spectrum using the neural mass model, which has a much less structured profile: see Fig. 2B. We emulated a neural mass prediction by setting the transit time *υ*_*ij*_ to zero. This effectively shrinks the cortical manifold to a point, because each layer sees distant inputs from other layers instantaneously. One can see this intuitively by noting that when the transit time is zero the temporal part of the connectivity kernel *d*_*ij*_(*x*, *t*) = *κ*_*ij*_(|*x*|)*δ*(*t*) does not depend upon distance. This precludes propagation delays from contributing to the dynamics.

The simulated spectral responses in Fig. 2 assumed spatially and temporally white inputs or innovations. Generally, however, these innovations themselves have frequency profiles, which we may not necessarily know. In the next section, we turn to a generative model for empirically observed spectra that contains not just synaptic and connectivity parameters but also parameters controlling the spectral profile of neuronal innovations and channel noise.

### Summary

In this section, we have reviewed neural field models that furnish predicted spectral responses to exogenous input as tractable functions of key synaptic and coupling parameters. We then considered briefly the more structured spectral density of these predictions that derives from considering spatial dynamics. The key observation here is that by including spatial dynamics on cortical manifold, we can account for the structured frequency responses in observed spectra. The example in Fig. 2 shows a fairly typical and simple form for realistic parameter values. We will see below that neural field models can produce much more complicated and exotic spectral profiles, depending upon the parameters chosen. It is this behavior that makes neural field models a potentially more plausible generative model of empirical observations, in relation to their neural mass homologues. In the next section, we consider how to use the neural field model above as an observation or generative model in dynamic causal modeling of steady-state responses.

## A neural-field DCM

In this section, we describe how the neural field model above can be embedded in a probabilistic model of empirical data. This can be regarded as an extension of dynamic causal modeling for steady-state responses (Moran et al., 2007, 2009); where we replace the conventional neural mass model with a neural field model. In this paper, we will focus on the role of intrinsic connections and consider a DCM of single-source data. As noted above, the equations of the previous section provide an analytic expression for the cross-spectral density between electrophysiological samples from a cortical source. These cross-spectra are parameterized completely in terms of unknown biophysical parameters describing the synaptic kinetics and intrinsic connectivity, given the input spectrum. In what follows, we describe how this mapping from free parameters to observed cross-spectra can be used to create an observation model (i.e., a dynamic causal model) of steady state responses. Although, in this paper, we deal only with real auto-spectra from single channels, we describe the more general procedure for inverting models of complex cross-spectra sampled by multiple channels.

### The generative model

To complete our specification of a generative model, we assume the observed cross-spectra **g**_*Y*_(*ω*) (denoted by boldface) are a mixture of predicted cross-spectra *g*_*Y*_(*ω*, *θ*), channel-noise *g*_*N*_(*ω*, *θ*)and Gaussian error *ε*(*ω*) (see (Moran et al., 2009) for details):

(30) gY(ω)=gY(ω,θ)+gN(ω,θ)+ε(ω)gY(ω,θ)=∑kL(k)T(k,ω)gU(k,ω)T(k,ω)*L(k)*gN(ω,θ)=αN+βNωgU(k,ω)=αU+βUωRe(ε)~N(0,∑(ω,λ))Im(ε)~N(0,∑(ω,λ)).

Note here that we have replaced the integral in Eq. (22) with a summation over discrete spatial frequencies (c.f., Eq. (29)). The spectra of channel noise *g*_*N*_(*ω*, *θ*), like those of the input *g*_*U*_(*ω*), are parameterized in terms of (unknown) white and pink components. We have assumed here that the input is spatially white. This means that the input spectrum does not depend upon spatial frequency. Eq. (30) provides the basis for our generative model and calls on extra free parameters controlling the spectra of the inputs and channel noise *θ* ⊂ {*α*_*N*_, *α*_*U*_, *β*_*N*_, *β*_*U*_}; and the amplitude of observation error *Σ*(*ω*, *λ*). Gaussian assumptions about the observation error mean that we have a probabilistic mapping from all unknown parameters to observed data features. Inversion of this model means estimating, probabilistically, the free parameters given data.

### Inverting models of complex data-features

Almost universally, the fitting or inversion of Dynamic Causal Models optimizes a free energy bound on the log-evidence for a model *m*. This bound is optimized with respect to a variational density *q*(*θ*) on the unknown model parameters. By construction, the free energy bound ensures that when the variational density maximizes free energy, it approximates the true posterior density over parameters, *q*(*θ*) ≈ *p*(*θ*|*y*, *m*). At the same time, the free energy itself F(y,q)≈lnp(y|m) approximates the log-evidence (marginal likelihood) of the data. The (approximate) conditional density and (approximate) log-evidence are used for inference on parameters and models respectively. In other words, one first compares different models (e.g., with and without particular connections) using their log-evidence and then turns to inferences on parameters, under the model selected.

One usually assumes the conditional density has a Gaussian form q(θ)=N(μ,C). This is known as the Laplace assumption. The conditional density is quantified by the most likely value of the parameters, *μ* and their conditional covariance *C* (inverse precision) that encodes uncertainty about the estimates and their conditional dependencies. To optimize the conditional mean and covariance, we need to express the free energy in terms of (generally) complex data-features like observed cross-spectra.

### The free energy of complex data-features

The free energy is a quantity which, by construction, is always greater than the log evidence above (by Gibbs inequality). It was introduced by Richard Feynman in the context of path integral formulations of quantum mechanics and has been used extensively in machine learning to finesse the difficult problem of exact Bayesian inference (by maximizing the free energy with respect to the parameters). It is called free energy because it comprises two terms: the first is an energy term, which is the log likelihood and prior of the data and model parameters, expected under the variational density. The second term is simply the entropy of the variational density. The free energy is simply the average of the log-likelihood and log-prior of the model, under the variational density and its entropy. For nonlinear models, under Gaussian assumptions about the variational density and observation noise, the free energy has a very simple form:

(31) F=G(μ)+12ln|∂μμG|G=−12Re(ε)TΣ−1Re(ε)−12Im(ε)TΣ−1Im(ε)−12ρTΩ−1ρ−12ln|Σ|−12ln|Ω|ε=gY(ω,μ)+gN(ω,μ)−gY(ω)ρ=μ−ϕ.

Here, *g*_*Y*_(*ω*, *μ*) + *g*_*N*_(*ω*, *μ*) are the predictions of the data features **g**_*Y*_(*ω*) and *ε*(*μ*) are the corresponding prediction errors with covariance *Σ*(*ω*, *λ*). Similarly, ρ(μ)∈R are prediction errors on the parameters, in relation to their prior density p(θ|m)=N(ϕ,Ω). Model complexity in Eq. (31) corresponds to the $-\frac{1}{2}\rho^{T}\Omega^{-1}\rho$ term: this reports the deviation of the estimated parameters from their prior expectations and effectively penalizes the free-energy objective function in proportion to the degrees of freedom used to explain the data.

For complex data, we have to separate the real and imaginary parts of the sum of the squared prediction error above. This is because the sum of an absolute value is not the absolute value of a sum. Similarly, the partial derivatives of the Gibb's energy G(μ), with respect to the parameters are separated into real and imaginary parts:

(32) ∂μG=−Re(∂με)TΣ−1Re(ε)−Im(∂με)TΣ−1Im(ε)−Ω−1ρ∂μμG=−Re(∂με)TΣ−1Re(∂με)−Im(∂με)TΣ−1Im(∂με)−Ω−1.

These gradients are used in a Gauss–Newton scheme to optimize the conditional mean and covariance iteratively, until the free energy has been maximized:

(33) $$\begin{array}{c} \mu =\underset{\mu }{\arg\max}F(\mu ,g_{Y}(\omega ))\\ C=-\partial_{\mu \mu }G{\left(\mu \right)}^{-1}\text{.} \end{array}$$

In practice, things are a little more complicated because one often makes a mean-field assumption when estimating parameters of the model *θ* and the error covariance, *Σ*(*ω*, *λ*). In other words, the precision (inverse covariance) of the observation error is usually assumed to be conditionally independent of the parameters. The gradient ascent then becomes a coordinate ascent that optimizes the conditional expectations of the model and error covariance parameters *λ* respectively. This is called Variational Laplace. A full description of these schemes can be found in (Friston et al., 2007).

### Summary

In this section, we have considered the central role of the free energy bound on log-evidence used in model selection and inversion. The only thing we have to worry about, when dealing with complex data, is to separate the real and imaginary parts of the data (and implicitly prediction errors), when evaluating the free energy and its gradients. Having done this, we can then use standard schemes to select among competing models to find the one that has the highest free energy (log-evidence). One can then examine the conditional parameter estimates of the selected model. In this paper, we will not be dealing with complex data because we will be considering the (real) auto-spectra from single channels. However, in more general applications, we have to consider the complex cross spectra among different channels. We now illustrate the application of this scheme and characterize the effects of key model parameters on predicted spectral responses.

## Model inversion and characterization

This section presents a statistical characterization of the model, in terms of its identifiability and changes in spectral predictions, with respect to key model parameters. In the context of DCM, similar analyses have been performed for various models of neural activity in Chen et al. (2008), Friston et al. (2003) and Moran et al. (2007, 2008). We will here compare and contrast the predictions of the neural field formulation with its neural mass equivalent, to illustrate the effects of spatial dynamics. We will use biophysically plausible priors for which the neural mass model is known to have a stable fixed point. Stability is guaranteed because the neural field model admits a stable solution during model inversion. To establish face validity and identifiability of the model, we used simulated data to ensure the inversion scheme was able to recover veridical estimates; and to ensure that model comparison using the log-evidence was able to identify the correct model. If at any point during model inversion we encountered a bifurcation from a stable fixed point, the inversion scheme automatically reverts to the previous parameter estimate. To quantify the effects of various parameters on the predictions, we examined the change in the spectral response with respect to each parameter. This can be regarded as a structural stability analysis, expanding around a particular set of parameter values. These values were obtained by inverting a neural field model using real LFP data. We describe the synthetic and empirical data and then report the results of model comparison, conditional estimates of model parameters and the effects of changing these parameters. For comparison, the analyses are repeated for both the neural field and neural mass formulations, where appropriate.

### Synthetic data and validation

We first examined the conditional mean of the transit time when the true log-scaling deviates from its prior expectation (of zero). This reveals how our model inversion operates in regions of parameter space that are remote from prior assumptions. We focused on the transit time because this parameter determines the contribution of spatially extended dynamics to observed temporal frequencies. Indeed, as we have seen above, in the limiting case that the transit time tends to zero (conduction speed tends to infinity), the neural field model reduces to a neural mass model.^2^ Furthermore, inferring axonal conduction speeds using single channel data illustrates our point in the introduction that suitably informed models enable one to access the spatial attributes of neuronal infrastructures, even in the absence of spatially resolved data.

**Fig. 3:**
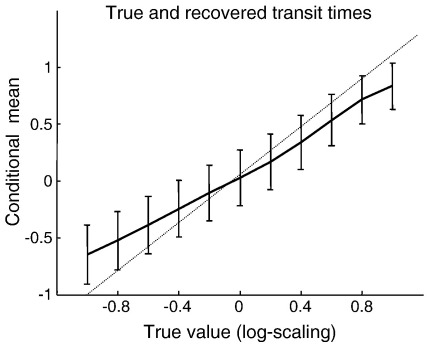
*Sensitivity analyses of synthetic data*. This figure shows the conditional estimates of the inverse conduction speed parameter, *υ*. These estimates were obtained from simulated data sets generated using *υ* as per Table 1, with a log-scaling from − 1 to 1. We illustrate the agreement between the true parameter value (dotted line) and its conditional estimate. The bars represent 90% conditional confidence intervals.

We synthesized observed spectra by setting the values of the parameters equal to their prior, with the exception of transit time, which was varied over a log-scaling range of − 1 to 1 (i.e. 36% to 272%). The synthetic data were generated according to Eq. (30) and inverted by optimizing the conditional mean and covariance as described in the previous section using Eq. (33). The resulting conditional estimates are shown in terms of the conditional mean and 90% confidence intervals in Fig. 3. One can see that there is a remarkable agreement between the conditional mean and true values and that the (relatively precise) confidence intervals include the true value (with the exception of the most extreme deviations). This is an interesting result that establishes the identifiability of the model, at least in relation to conduction speed. Clearly this only establishes face validity (the inversion does what it is meant to) and does not speak to the physiological validity of the model, which has to be addressed using empirical data (see below). Having said this, this sort of validation speaks to the possibility of estimating small differences in conduction velocity that might be elicited experimentally through pharmacological or other manipulations.

We then turned to inference on models and asked whether Bayesian model selection (Penny et al., 2010) could disambiguate between data generated by neural mass and neural field models. To do this, we simulated data using prior parameter values under neural field (transit time of 3 s per meter) and mass (a transit time of zero) assumptions and inverted both datasets using neural field and mass models. Table 2 shows the relative log-evidence of the four model inversions; as expected, we see that the neural field model is a better explanation for the data generated by neural fields, while the neural mass data would be better explained by neural mass models. The difference in log-evidence reflects the identifiability of the models: it can be seen from Table 1 that there are profound differences between the two models and of both data types in the direction anticipated. Interestingly, when the data were generated under neural field assumptions, the neural field model had much greater evidence than the corresponding neural mass model (with a log-evidence difference of over 100). Conversely, when the data were generated under neural mass assumptions, the log-evidence difference was only about 10. This suggests that the neural field model can explain spectra, even when spatial dynamics are suppressed. This is intuitively sensible, because the neural mass model is a special case of the neural field model. Generally, a log-evidence difference of three or more can be taken as strong evidence for one model over another (Kass and Rafter*y*, 1995). Quantitatively, these sorts of validations are important when it comes to interpreting model comparisons using empirical data, which we turn to next.

### Empirical LFP data

Local field potentials were recorded from primary (A1) and secondary (A2) auditory cortex in the Lister hooded rat, following the application of the anesthetic agent Isoflurane; 1.4 mg (see Moran et al., 2011 for details). In brief, we used a telemetric recording system (TSE Systems) with chronically implanted epidural Silverball electrodes above the auditory cortex. During data acquisition, acoustic white noise stimuli at a level of 83 dB (sampling rate 25 kHz) and were delivered by an RX6 processor and two free field magnetic speakers (Tucker Davis Technologies, TDT) that were placed with a distance of 15 cm, on both sides of the rat's head.

Telemetric LFP recordings were acquired using DasyLab (Version 9.0, 2005, National Instruments) at a sampling rate of 2 kHz. Filtering was applied online, integrated into the telemetry system (0.6–60 Hz). Ten minutes of recordings were extracted from the continuous time domain data and down-sampled to a sampling rate of 125 Hz. Frequency domain data-features **g**_*Y*_(*ω*) were obtained from this epoch using a vector autoregression model of order eight (using the SPM Spectral Toolbox: http://www.fil.ion.ucl.ac.uk, (Roberts and Penny, 2002)). Here, we focus on the spectral response in A1.

### DCM and model inversion

There exists a vast literature on comparing neural field predictions with spectra (see e.g. Atay and Hutt, 2006; Breakspear et al., 2006; Freeman, 2003,2005; Golomb and Amitai, 1997; Jirsa, 2009; Liley et al., 2002; Nunez, 1995; Robinson et al., 2001, 2002, 2003, 2004, 2005; Robinson, 2006). Here, there is only one data channel and therefore our electromagnetic forward model reduces to a lead field over the assumed cortical source. We parameterized the lead field in terms of its amplitude and width, assuming that it had a Gaussian form as in Eq. (27). We then optimized the parameters of the neural field model using the Variational Laplace scheme described above. This model inversion used the prior expectations on the parameters in Table 1. We then repeated the model inversion but setting the inverse conduction velocity to zero. As above, this furnishes parameter estimates under a neural mass model that discounts the contribution of spatial dynamics.

**Fig. 4:**
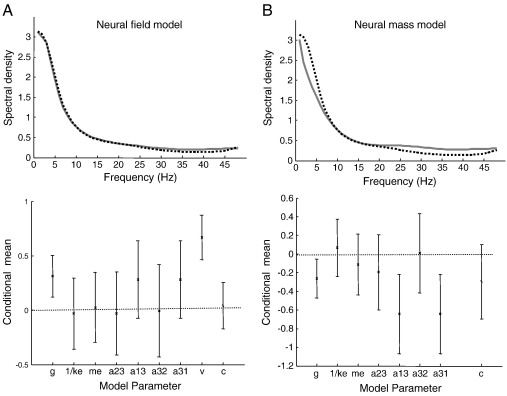
*Model inversion using spectra from rat auditory cortex*. A: Upper panel: real data (dashed line) and model predictions (full line) showing a 1/*ω* spectral profile that is typically seen under anesthesia. The lower panel shows the conditional estimates (90% confidence intervals) of the neural field model parameters using empirical data from a single trial recording of auditory responses in the rat. The estimates lie close to the prior values (a log-scaling of zero), which were motivated by animal physiological studies (Jansen and Rit, 1995). B: Neural mass model estimates of the same data exhibit a similar fit (upper panel) and conditional estimates (lower panel)**.**

The observed and predicted auto-spectra for the neural field and mass models are shown in the upper panels of Fig. 4. These model predictions illustrate nicely the difference between the field and mass models: one can see that the neural field model has approximated the preponderance of low frequencies more accurately than the neural mass model. This is because it has extra degrees of freedom; namely conduction velocity and the extent of lateral connections. These extend the repertoire of predictions to include those afforded by spatial dynamics. Crucially, the log-evidence for the neural field model was 1271 above the log-evidence for the neural mass model. This suggests that there is a very strong evidence for spatial dynamics over the cortical manifold in these auditory cortex data.

The conditional estimates of the log-scaling of the model parameters are shown in the lower panels of Fig. 4. Fig. 4A shows the conditional expectations of the neural field model parameters and their 90% confidence intervals (bars). Note that most of the confidence intervals include a log-scaling of zero. This is not surprising because the prior values that are scaled were chosen carefully to reproduce spectra that are typical of this recording setup. The two exceptions were transit time and gain that increased by 160 and 120% over their prior values respectively. The corresponding conditional estimates for the neural mass model are shown in Fig. 4B. These illustrate the similarities and differences between the inferred synaptic parameters and intrinsic connection strengths between the neural field and mass models: most parameter estimates concur between the two formulations, with the exception of the synaptic strengths between interneurons and pyramidal cells and the extent of lateral connections. Notice that the spatial extent parameter can still be estimated with a degree of precision under the neural mass model. This is because it is an integral part of the strength of intrinsic connections (see Eq. (25)).

Obtaining parameter estimates from observed spectra is an important endeavor and several authors have suggested relevant schemes using both neural field and neural mass models (Daunizeau et al., 2009; Galka et al., 2008; Riera et al., 2007; Rowe et al., 2004; Schiff and Sauer, 2008; Valdes et al., 1999; van Albada et al., 2010). It is interesting to note that we have here formulated a neural mass model as a limiting case of a neural field model by simply applying very precise shrinkage priors to the conduction velocity. This provides a useful perspective on the relationship between these two models, in terms of the implicit assumptions we make when modeling observed data. A pragmatic advantage of emulating neural mass models with a transit time of zero is that we can simply apply precise shrinkage priors (to transit time) to facilitate model comparison. In other words, it provides a simple means of comparing models with and without spatial dynamics. The inversions above are simply intended to demonstrate the face validity of the approach: namely that veridical models and plausible parameter estimates can be recovered. Clearly, we are assuming here that the cortical dynamics recorded by our local field potential electrode are due to activity on a cortical manifold. In future work, we will address whether model selection and parameter estimates concur with independent manipulations of synaptic and spatial parameters.

### Characterizing the effect of changes in model parameters

**Fig. 5:**
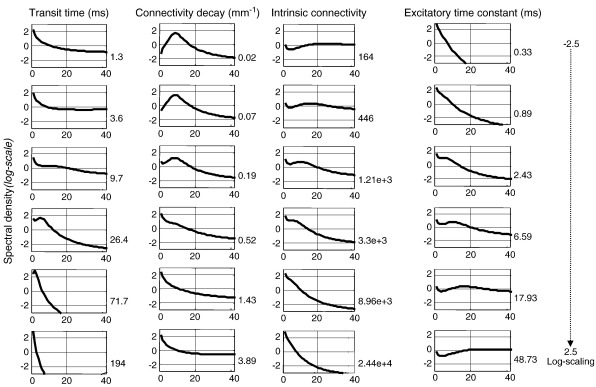
*Sensitivity of model predictions*. This figure shows semilog plots of the changes in the spectral profile of the cortical sources while varying the transit time, lateral extent of intrinsic connectivity, excitatory synaptic time constant and the strength of intrinsic connections between interneurons and pyramidal cells over a log-scaling range of − 2.5 to 2.5 (i.e. 8% to 1200% from top to the bottom). We include the values of the parameters in the lower right corner of each plot.

In our final analysis, we characterized the variations in spectral profile produced by changes in the parameters about the conditional means from the empirical data above. This analysis recovers some of the results relating model parameters to low-pass cutoffs in the spectrum that have been obtained in systematic earlier work (Nunez, 1995; Nunez and Srinivasan, 2006; Robinson et al., 2001, 2002, 2003; Valdes-Sosa et al., 2009) and is here given for illustration purposes only: Fig. 5 shows the change in predicted spectra, as a function of frequency, while varying the transit time, connectivity extent, excitatory synaptic time constant and intrinsic synaptic coupling strength between interneurons and pyramidal cells over a log-scaling range of − 2.5 to 2.5 (i.e. 8% to 1200%). The key thing to notice here is that the changes show a complicated frequency dependency. This is the latitude that neural field models have, over neural mass models, when fitting empirical spectra. Furthermore, we observe quantitatively different behaviors for different parameter regimes: when decreasing the velocity, new spectral peaks appear and lower frequencies predominate. Conversely, when transit time is small (velocity increases) the spectra exhibit the simple 1/*ω* profile characteristic of the neural mass model. Similarly, the field spectra resemble those of a neural mass when decreasing the range of lateral connections (increasing *c*). As expected, an increase of the excitatory synaptic time constant (decrease in the constant *κ*_*e*_) enhances low frequency alpha peaks in the spectra with a progressive shift towards beta frequencies. The effect of increasing the strength of intrinsic connections between the inhibitory interneurons and pyramidal cells *a*_13_ is similar to the effect of decreasing their extent: the high frequency structure of neuronal dynamics is lost and the peaked shape of the spectra disappears so that it resembles a neural mass model.

### Summary

In summary, we have illustrated model inversion using a multilayered neural field model describing a single cortical manifold or source. Conceptually, this analysis demonstrates that conventional neural mass models can be regarded as a special case of a more general neural field formulation. This special case obtains when we preclude spatial dynamics by imposing prior beliefs on the spatial parameters (conduction velocity is infinite and the transit time becomes vanishingly small in relation to the timescale of neural activity). The second key point to be taken from this section is that we were able to obtain fairly precise estimates of spatial parameters like conduction velocity, despite the fact we only have a single electrode (see also Rowe et al., 2004; Robinson et al., 2004; van Albada et al., 2010). This speaks to the fact that using informed and plausible generative models of data can sometimes help access hidden states and parameters that are not observed directly. In this case, our prior assumptions about the spatial form of cortical activity allowed us to make quantitative inferences about the speed of lateral cortical interactions, despite having no spatial information in the data. Finally, we have provided proof of principle that Bayesian model selection can distinguish between neural field and mass formulations of cortical dynamics and have presented a simple example suggesting that field models provide better explanations of empirical data.

## Conclusion

In recent years, several people have promoted the use of neural models to characterize neuronal dynamics. For example, neural mass models have been inverted explicitly using appropriate Kalman filters; e.g. (Riera et al., 2007; Valdes et al., 1999). Furthermore, the inverse problem for a single population described by a neural field equation has been addressed in Daunizeau et al. (2009), Galka et al. (2008), Schiff and Sauer (2008). Daunizeau et al. replaced the standard dipole source used in neural mass models with the principal Fourier mode of a neural field model with exponentially decaying synaptic density; this corresponds to a standing wave with temporal dynamics identical to those of neural mass models. Schiff and Sauer also estimated spatiotemporal neuronal activity via the elegant application of an unscented Kalman filter, while Galka et al. used a similar maximum likelihood framework to invert neural field models of EEG data. In this paper, we hope to have further demonstrated the usefulness of neural field models in the context of dynamic causal modeling. We conclude by considering the next steps in elaborating neural field models of distributed cortical responses.

### Models of multiple sources

In this introductory paper, we consider only the spectrum from a single cortical source as observed, through local field potentials. In a subsequent paper, we will extend the model to cover not just intrinsic connections, but also extrinsic connections among cortical sources. This becomes a bit more complicated because the extrinsic connectivity kernels (and associated transfer functions) no longer have the same form used for intrinsic connectivity. This is because there is a long and finite delay associated with extrinsic connections that does not depend upon the position within the manifold associated with each source (Brackley and Turner, 2009; Jirsa and Kelso, 2000; Qubbaj and Jirsa, 2009; Robinson et al., 2002). In this context, we have to make a distinction between extrinsic and intrinsic connectivity kernels *d*_*ij*_^(*ab*)^(*x*, *t*) within and between sources. Here *d*_*ij*_^(*ab*)^(*x*, *t*) denotes the connectivity kernels from the *j*-th layer of source *b* to the *i*-the layer of source *a*; where

(34) $$d_{ij}^{\left(ab\right)}(x,t)=\{\begin{array}{ll} \kappa_{ij}^{\left(ab\right)}(|x|)\delta (t-\upsilon_{ij}|x|) & a=b\\ \kappa_{ij}^{\left(ab\right)}(|x|)\delta (t-\Delta_{\mathrm{ab}}) & a\neq b \end{array}\text{.}$$

Here, $\Delta_{\mathrm{ab}}$ is the conduction delay between two distinct sources. Note that this model of extrinsic connectivity still accounts for the spatial location of coupling between sources and allows for divergence of extrinsic connections in the same way that intrinsic connections have a lateral dispersion. However, the conduction delay of extrinsic connections is fixed and does not depend on the location within the layers of each source. We will develop this model in a subsequent paper and consider it in the context of non-invasive EEG and MEG.

### Modeling invasive data

The second application, that we hope to pursue, concerns spatially resolved electrophysiological measurements. The idea here would be to use a generative model that considers a single cortical manifold but exploit the fact that the dynamics on this manifold are sampled by multiple electrophysiological or optical sensors. This calls for a more detailed consideration of the lead fields associated with each sensor and the way that electrodes sample local electromagnetic responses over space. This represents an intriguing inverse problem that can, in principle, be solved by inverting an appropriate DCM of the sort described above. In this setting, it may be the case that the parameters governing not just the spatial aspects of cortical microcircuitry but also the spatial characteristics of the lead fields (i.e., sensitivity profiles) have to be estimated.

## Figures and Tables

**Table 1:** Prior expectations of model parameters.

Parameter	Physiological interpretation	Prior mean: P
*m*_*e*_, *m*_*i*_	Maximum postsynaptic depolarization	8, 32 (mV)^a^
*κ*_*e*_, *κ*_*i*_	Postsynaptic time constants	1/4, 1/28 (ms^−1^)^a^
*α*_13_, *α*_23_, *α*_31_, *α*_32_	Amplitude of intrinsic connectivity kernels	2000, 8000, 2000, 1000
*c*_*ij*_	Intrinsic connectivity decay constant	0.32 (mm^−1^)^b^
*r*, *η*, *g*	Sigmoid parameters (post synaptic firing rate function)	0.54, 0, 0.135^a^
*s*_*ij*_	Conduction velocity	3 m/s^b^
ℓ	Radius of cortical source	50 (mm)^b^

In practice, these priors are scaled by log-scale parameters with a prior mean of zero (and precisions of sixteen) to ensure positivity.

a Wendling, et al. (2000).

b Kandel, et al. (2000).

**Table 2:** Log-evidence of neural models.

Model	Neural field model	Neural mass model
Neural field data	167.39	− 35.21
Neural mass data	160.51	170.82

This table presents the log-evidence for neural field and mass models, using synthetic data generated by the respective models (at the prior expectations of their parameters in Table 1).

## Appendix

Here, we show that Eqs. (16) and (25) imply Eq. (24). Let us define

(A1) $$\begin{array}{c} \mu_{1}=\iint d_{13}(x-x^{\prime },t-t^{\prime })\sigma (v_{3}(x^{\prime },t^{\prime }))dx^{\prime }dt^{\prime }\\ \mu_{2}=\iint d_{23}(x-x^{\prime },t-t^{\prime })\sigma (v_{3}(x^{\prime },t^{\prime }))dx^{\prime }dt^{\prime }\\ \mu_{3}=\iint \left(d_{31}(x-x^{\prime },t-t^{\prime })\sigma (v_{1}(x^{\prime },t^{\prime }))-d_{32}(x-x^{\prime },t-t^{\prime })\sigma (v_{2}(x^{\prime },t^{\prime }))\right)dx^{\prime }dt^{\prime }\text{.} \end{array}$$

Then taking the Fourier transforms of both sides of the above equations, we find

(A2) $$\begin{array}{c} M_{1}(k,\omega )=D_{13}(k,\omega )\Sigma (v_{3})(k,\omega )\\ M_{2}(k,\omega )=D_{23}(k,\omega )\Sigma (v_{3})(k,\omega )\\ M_{3}(k,\omega )=D_{31}(k,\omega )\Sigma (v_{3})(k,\omega )-D_{32}(k,\omega )\Sigma (v_{2})(k,\omega )\text{.} \end{array}$$

We now focus on Eq. (A2c) (the remaining two equations can be treated similarly). Substituting

(A3) $$\begin{array}{l} D_{31}(k,\omega )=D_{32}(k,\omega )=\frac{\alpha_{31}(c_{31}+i\upsilon \omega )}{c_{31}^{2}-\upsilon_{}^{2}\omega^{2}+2i\upsilon c_{31}\omega +k^{2}}\\ \; \end{array}$$

in Eq. (A2c) we obtain

(A4) $$(c_{31}^{2}-\upsilon^{2}\omega^{2}+2i\upsilon c_{31}\omega +k^{2})M_{3}(k,\omega )=\alpha_{31}(c_{31}+i\upsilon \omega )(\sum (v_{1})-\sum (v_{3}))(k,\omega )\text{.}$$

Taking the inverse Fourier transform of the above equation and multiplying the resulting equation by *s*^2^, we find Eq. (24f). Similarly for Eq. (24d,e).

## Footnotes

1 The existence of a steady-state follows from the theory of integral equations. A linear stability analysis can be carried out following the pioneering work of Wilson and Cowan, 1973 (see also Coombes, 2005; Jirsa, 2009; Pinotsis and Friston, 2011) who considered the neural field equation as a system exhibiting spontaneous pattern formation.

2 In taking the limit *s* → *∞* in Eq. (24) the spatial derivatives vanish as the cortical manifold shrinks (or the neural field stretches) and depolarization becomes spatially homogeneous. In this limit, the corresponding electrophysiological predictions effectively coincide with those generated by a neural mass model.
